# Knock out of the *PHOSPHATE 2* Gene *TaPHO2-A1* Improves Phosphorus Uptake and Grain Yield under Low Phosphorus Conditions in Common Wheat

**DOI:** 10.1038/srep29850

**Published:** 2016-07-15

**Authors:** Xiang Ouyang, Xia Hong, Xueqiang Zhao, Wei Zhang, Xue He, Wenying Ma, Wan Teng, Yiping Tong

**Affiliations:** 1 State Key Laboratory for Plant Cell and Chromosome Engineering, Institute of Genetics and Developmental Sciences, Chinese Academy of Sciences, Beijing 100101, China; 2 Taizhou Academy of Agricultural Sciences, Linhai, Zhejiang 317000, China

## Abstract

MiR399 and its target *PHOSPHATE2 (PHO2*) play pivotal roles in phosphate signaling in plants. Loss of function mutation in *PHO2* leads to excessive Pi accumulation in shoots and growth retardation in diploid plants like *Arabidopsis thaliana* and rice (*Oryza sativa*). Here we isolated three *PHO2* homologous genes *TaPHO2-A1*, -*B1* and -*D1* from hexaploid wheat (*Triticum aestivum*). These *TaPHO2* genes all contained miR399-binding sites and were able to be degraded by tae-miR399. *TaPHO2-D1* was expressed much more abundantly than *TaPHO2-A1* and -*B1*. The ion beam-induced deletion mutants were used to analyze the effects of *TaPHO2s* on phosphorus uptake and plant growth. The *tapho2-a1, tapho2-b1* and *tapho2-d1* mutants all had significant higher leaf Pi concentrations than did the wild type, with *tapho2-d1* having the strongest effect, and *tapho2-b1* the weakest. Two consecutive field experiments showed that knocking out *TaPHO2-D1* reduced plant height and grain yield under both low and high phosphorus conditions. However, knocking out *TaPHO2-A1* significantly increased phosphorus uptake and grain yield under low phosphorus conditions, with no adverse effect on grain yield under high phosphorus conditions. Our results indicated that *TaPHO2s* involved in phosphorus uptake and translocation, and molecular engineering *TaPHO2* shows potential in improving wheat yield with less phosphorus fertilizer.

Phosphorus (P) is one of the three macronutrients essential for plant growth and reproduction, and phosphate (Pi) is often non-available to plants because of the low abundance and immobile in soils. Therefore, P fertilizers are often required for high yield of crops in modern agriculture. However, due to the high fixation and low diffusion rate in most soils, no more than 30% of the applied P is used by the cultivated plants^1^. The remains results in eutrophication and nonrenewable phosphate rock waste. Thus improving P use efficiency (PUE) in crops becomes great importance in ensuring sustainable development of agriculture. Exploring the molecular mechanisms in regulation of P uptake and utilization may help us to breed wheat with improved PUE.

Plants have evolved complicated physiological and biochemical responses to adapt to the limiting P conditions. The molecular mechanisms regulating these responses have been well documented in the model plants *Arabidopsis thaliana* and rice (*Oryza sativa*)^2^,^3^. PHOSPHATE STARVATION RESPONSE 1 (PHR1) in Arabidopsis, a MYB-CC type transcription factor, plays a key role in regulating the expression of Pi starvation-induced (PSI) genes by binding to P1BS (PHR1 binding site) cis-element with an imperfect palindromic sequence^4^. Overexpression of *PHR1* and its homologs activates the expression of many PSI genes including Pi transporters, *Phosphate Starvation1 (IPS1*) and miR399, and leads to excessive Pi accumulation in shoots of Arabidopsis, rice and wheat (*Triticum aestivum*)^5^,^6^,^7^,^8^,^9^,^10^. MiR399 is the first reported microRNA specifically induced by Pi starvation^11^. Under Pi deficiency conditions, miR399 is upregulated by PHR1, and then reciprocal downregulates the transcript level of *Phosphate 2 (PHO2*) which contains multiple miR399 target sites in the 5′-UTR (untranslated region)^11^,^12^,^13^. However, the miR399 activity in cleaving *PHO2* is regulated by *IPS1* according to the target mimicry mechanism^14^. *PHO2*, encoding an ubiquitin-conjugating E2 enzyme (UBC24), negatively regulates Pi uptake and root-to-shoot translocation. Loss of function of *PHO2* and overexpression of miR399 both result in Pi over accumulation in shoots^13^,^15^,^16^. It has been reported that the expression of several Pi transporters were increased in *pho2* mutants and miR399-overexpression plants, such as *AtPHT1.8* and *1.9* in Arabidopsis^12^,^13^, and *OsPT1, 2, 4* and *8* in rice^16^. Further analysis shows that *PHO2* genetically interacts with *PHOSPHATE 1 (PHO1*) and *PHT1.1*^17^,^18^. *PHO1*, encoding an integral membrane protein, is involved in Pi transfer from roots to shoots^19^,^20^. The ubiquitination of PHO1 mediated by PHO2 demonstrates that PHO1 is the direct downstream component of PHO2^17^. Several high affinity Pi transporters (PHT1s) were also identified as downstream of PHO2 by using an iTRAQ (for isobaric tags for relative and absolute quantitation)- based quantitative membrane proteomic method^18^. PHO2 is found to mediate PHT1 proteins degradation, and loss of function of *PHT1.1* alleviates the Pi toxicity phenotype displayed by *pho2* in Arabidopsis^18^. These results suggest that PHT1s act as downstream components of PHO2. Taking together, PHO2 plays a pivotal role in Pi homeostasis regulation by coordinating the activities of Pi uptake and roots to shoots Pi translocation through PHT1s and PHO1^18^,^21^,^22^.

Wheat is one of the most important food crops, and wheat production consumed 16.1% of the phosphate fertilizer, higher than maize (*Zea mays*, 15.2%), rice (12.8%) and other cereals (4.4%)^23^. Therefore, improving PUE of wheat is important in sustainable use of P resources. Understanding the molecular mechanisms regulating P use may facilitate the breeding of wheat with improved PUE. For example, Wang *et al*.^8^ proposed that *TaPHR1* involves in Pi signaling in wheat. Overexpressing *TaPHR1* in wheat up-regulates a subset of PSI genes, stimulates lateral root branching, improves Pi uptake and grain yield^8^. Overexpression of the Pi transporters *TaPT2*^24^ and *TaPHT1.4*^25^ both enchance Pi uptake and plant developmenmt in wheat. Overexpression of the β-expansin gene *TaEXPB23* increased lateral root number in transgenic tobacco (*Nicotiana tabacum*) plants under excess-P and low-P conditions^26^. By comparing the transcriptome profiles of wheat and rice, an IPS1-mediated signaling cascade (include PHR1-IPS1-miR399-PHO2) and its downstream functions involved in a general response to Pi starvation were revealed^27^. However, compared with Arabidopsis and rice, molecular mechanisms underlying Pi signaling are still largely unknown in wheat.

Here we cloned three PHO2 homologous genes *TaPHO2-A1, B1* and *D1* from wheat. The ion beam-induced deletion mutants of these *TaPHO2s* each showed higher expression of *TaPHO1* and *TaPHT1s*, and higher Pi concentrations in shoots than did the wild type. Field experiments exhibited that the *tapho2-a1* mutant displayed higher aerial P accumulation and grain yield than did the wild type under low P conditions. These results indicated that *TaPHO2* involved in Pi signaling and showed potential in improving PUE and yield in wheat.

## Results

### Identification of *PHO2* genes in wheat

**Figure 1:**
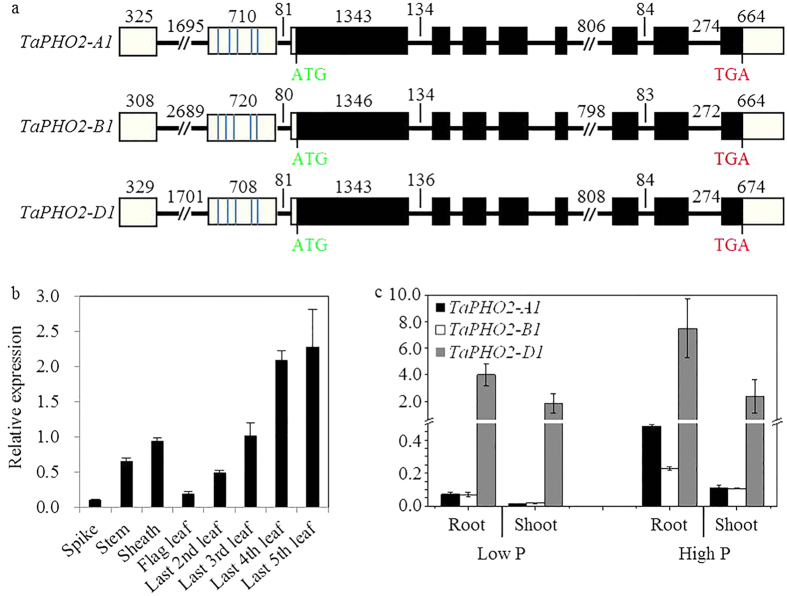
Gene structures and expression of the *TaPHO2s*. (**a**) The gene structures of *TaPHO2-A1, B1* and *D1*. The black boxes indicate the ORF of *TaPHO2s*; the white boxes indicate the UTR regions; the black lines indicate the introns. The blue ticks in the second exon depict the position of five putative miR399 binding sites. Numbers depict the length of exons or introns in the corresponding region. ATG, the start codon; TGA, the stop codon. (**b**) Overall relative expression levels of *TaPHO2* in the spikes, stems, leaf sheaths, flag leaves and top 2~5 leaves (from top to the bottom of the wheat plant) of the field grown wheat plants at the flowering stage. Error bars indicate SE (n = 5). (**c**) Relative expression levels of *TaPHO2*–*A1, B1* and *D1* in roots and shoots of the plants under 10 μM Pi (low P) and 200 μM Pi (high P) conditions at the seedling stage. Error bars indicate SE (n = 3).

We isolated the full-length cDNA and genomic DNA sequences of three *PHO2* homologues from the winter wheat variety Xiaoyan 81 by rapid amplification of cDNA ends (RACE) and genomic PCR amplification. These three *TaPHO2* genes were mapped on chromosomes 1A, 1B and 1D by using the Chinese Spring deletion lines (Supplemental Fig. S1), and were named as *TaPHO2-A1, TaPHO2-B1* and *TaPHO2-D1*, respectively. These three *TaPHO2* sequences shared highly sequence similarity in the open reading region, but had large variations in the introns, 5′ and 3′-UTRs. Each of them contained 10 exons (including 2 untranslated exons in the 5′-UTR), and had five putative miR399-binding sites in the second exon (Fig. 1a). The deduced protein sequences of these *TaPHO2* genes had conserved ubiquitin-conjugating catalytic (UBCc) domain at the C terminus (Supplemental Fig. S2). Phylogenetic analysis revealed that the three TaPHO2s belonged to the same subgroup with OsPHO2 from rice, and were more closely related to HvPHO2 from barley (*Hordeum vulgare*, Supplemental Fig. S3).

### Expression profiles of *TaPHO2s*

We investigated the spatial-temporal expression pattern of *TaPHO2* genes in different organs of the field-grown wheat plants at flowering stage. The *TaPHO2* transcripts were ubiquitously expressed in all the examined organs including spikes, stems, sheaths and leaves (Fig. 1b). We then analyzed the expression of *TaPHO2-A1, -B1* and *-D1* in roots and shoots of the wheat plants grown in nutrient solution at seedling stage. All the three *TaPHO2* genes had higher expression level in roots than in shoots (Fig. 1c). *TaPHO2-D1* displayed much higher transcript abundance than *TaPHO2-A1* and *-B1* did (Fig. 1c), indicating that *TaPHO2-D1* was possibly the primary member of *TaPHO2* in wheat. In both roots and shoots, the expression of the three *TaPHO2* genes was lower under low P conditions than that under high P conditions (Fig. 1c), suggesting that *TaPHO2* was down-regulated by Pi-deficiency.

### Regulation of *TaPHO2* by tae-miR399 and *TaIPS1*

**Figure 2:**
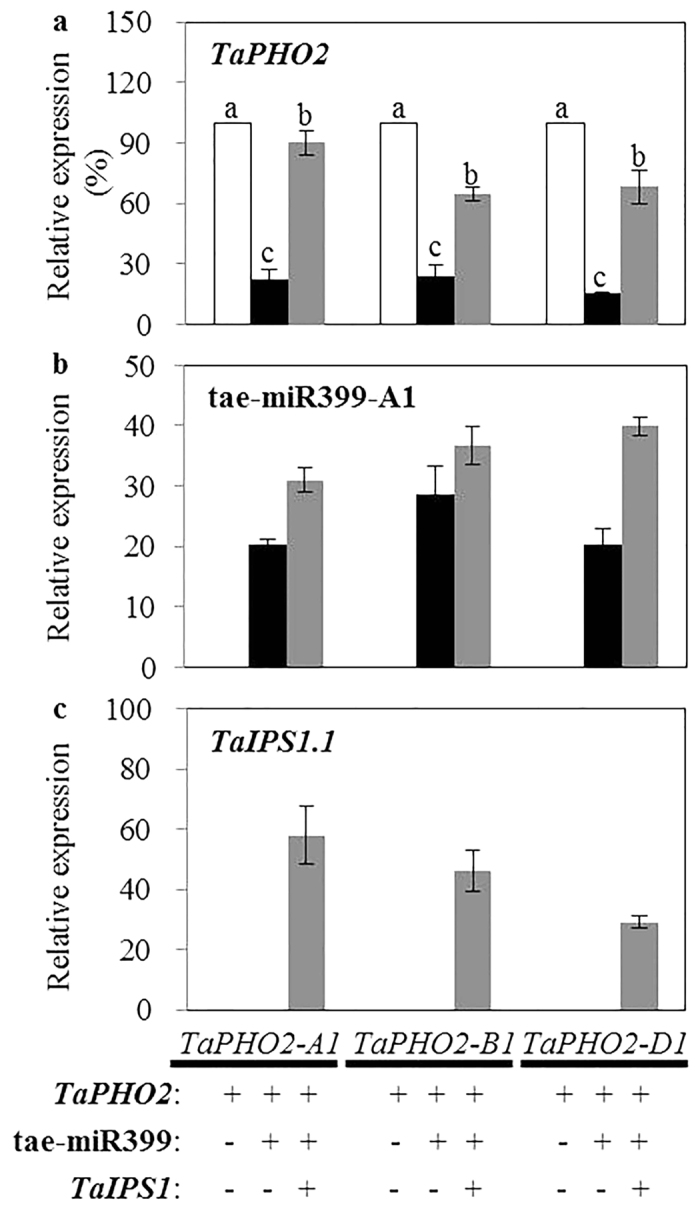
The regulation of the *TaPHO2* transcripts by tae-miR399 and *TaIPS1*. The relative expression levels of *TaPHO2* (**a**), tae-miR399-A1 (**b**) and *TaIPS1* (**c**) in the tobacco leaves transiently overexpressed the indicated gene(s). The expression of *TaPHO2* was presented as percentage of that in the control leaves which were transformed with *TaPHO2-A1, -B1* or *-D1* alone, the relative expression levels of tae-miR399-A1 and *TaIPS1* were normalized using the expression of *NtACTIN*. Different letters in (**a**) indicate significant difference at P < 0.05 level by Student’s *t* test. Error bars indicate SE (n = 6).

Sequence analysis revealed that there were five putative miR399-binding sites in 5′-UTRs of the three *TaPHO2* genes (Supplemental Fig. S4). In order to investigate whether *TaPHO2* could be degraded by tae-miR399, we used tobacco transient expression system to analyze this possibility. When *TaPHO2-A1, -B1* or *-D1* was co-transformed with tae-miR399-A1 in the tobacco leaves, the mRNA levels of all three *TaPHO2* genes were significantly lower than that transformed with *TaPHO2* gene alone (Fig. 2a). Moreover, sequence analysis of *TaIPS1s* also found that three *TaIPS1* genes all contained a motif with sequence complementarity to tae-miR399 (Supplemental Fig. S4). As all the three *TaIPS1* genes conferred the conserved complementary sequences with tae-miR399, only *TaIPS1.1* was chosen to check if it affected the degradation of *TaPHO2-A1, -B1* or *-D1* by tae-miR399. *TaPHO2* mRNA levels in the tobacco leaves transformed with *TaPHO2*, tae-miR399-A1 and *TaIPS1.1* were higher than that transformed with *TaPHO2* and tae-miR399-A1 (Fig. 2a). These results indicated that all the three *TaPHO2* genes were able to be degraded by tae-miR399, and *TaIPS1.1* inhibited this degradation.

### Plant growth and Pi distribution of *TaPHO2* deletion mutants in hydroponic culture

**Figure 3:**
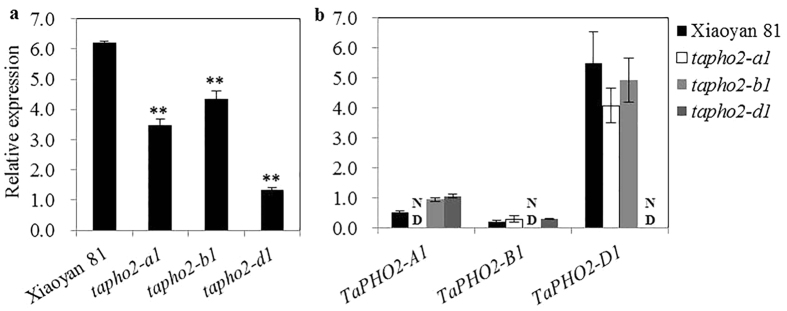
Relative expression levels of *TaPHO2* genes in roots of the wild type and *tapho2* mutant plants at seedling stage. The plants were grown in the nutrient solution containing 20 μM Pi, and the roots were used for gene expression analysis. (**a**) Overall relative expression levels of *TaPHO2s*; (**b**) Relative expression levels of *TaPHO2-A1, TaPHO2-B1* and *TaPHO2-D1*. Error bars indicate SE (n = 5). ND, not detectable. Asterisks indicate the significance of differences between wild type and *tapho2* mutants as determined by Student’s *t* test analysis: **P < 0.01, *P < 0.05.

To investigate the function of *TaPHO2* in wheat, we screened for the *TaPHO2* deletion mutants from the ion beam-induced mutants of variety Xiaoyan 81. After screening, the homozygous *tapho2-a1, b1* and *d1* mutants were obtained by ABI 3730 analysis (Supplemental Fig. S5). Before further analysis, three homozygous *tapho2* mutants were backcrossed twice with their wild type progenitor Xiaoyan 81, and homozygous BC_2_F_3_ or BC_2_F_4_ mutants were used for further analysis. Compared to the wild type plants, the overall *TaPHO2* expression levels were significantly declined (Fig. 3a). The *tapho2-d1* mutant had lower *TaPHO2* expression than did the *tapho2-a1*, and *tapho2-b1* mutants (Fig. 3a). We also did not detect the transcript of *TaPHO2-A1, -B1* and *-D1* in their corresponding mutant (Fig. 3b), indicating that *TaPHO2-A1, -B1* and *-D1* were deleted in their corresponding mutant.

**Figure 4:**
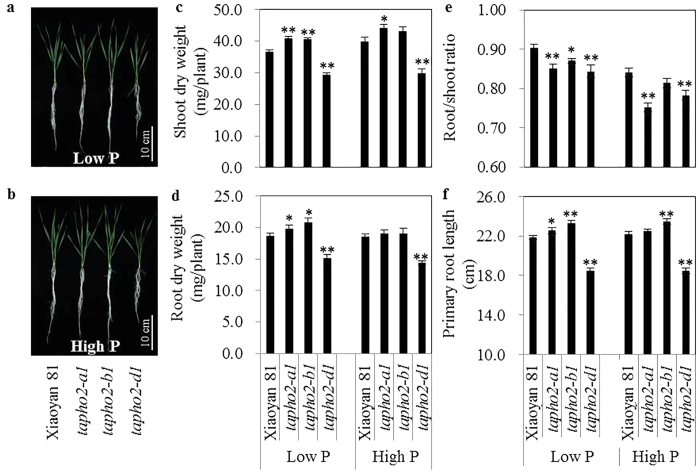
Growth performance of wild type and *tapho2* mutant plants grown in the nutrient solutions containing 10 μM Pi (low P) and 200 μM Pi (high P) at the seedling stage. (**a**,**b**) Images of the plants under low P (**a**) and high P (**b**) conditions. Scale bar = 10 cm; (**c**) Shoot dry weight; (**d**) Root dry weight; (**e**) Root/shoot dry weight ratio; (**f**) Primary root length. Error bars indicate SE (n = 5). Asterisks indicate the significance of differences between wild type and *tapho2* mutant plants as determined by Student’s *t* test analysis: **P < 0.01, *P < 0.05.

We evaluated the effects of deleting *TaPHO2* genes on the growth of wheat seedlings under low P and high P conditions in a hydroponic culture. Under low P conditions, the *tapho2-a1* and *tapho2-b1* mutants showed significantly higher shoot dry weight (SDW, Fig. 4c), higher root dry weight (RDW, Fig. 4d), lower root/shoot ratio (Fig. 4e), and longer primary root length (Fig. 4f) than did the wild type. Under high P conditions, the *tapho2-a1* mutant had significantly higher SDW (Fig. 4c) and lower root/shoot ratio (Fig. 4e) than did the wild type, and *tapho2-b1* mutant had longer primary root length (Fig. 4f) than did the wild type. Under both low P and high P conditions, the *tapho2-d1* mutant had significantly lower SDW, RDW, root/shoot ratio and shorter primary root length than did the wild type (Fig. 4c–f). These results suggested that the seedlings of the *tapho2-a1* and *tapho2-b1* mutants had advanced adaptive capacity to Pi-deficiency conditions, while significant repression of plant growth occurred in the seedlings of *tapho2-d1* mutant under both low P and high P conditions when the plants were grown in nutrient solution.

**Figure 5:**
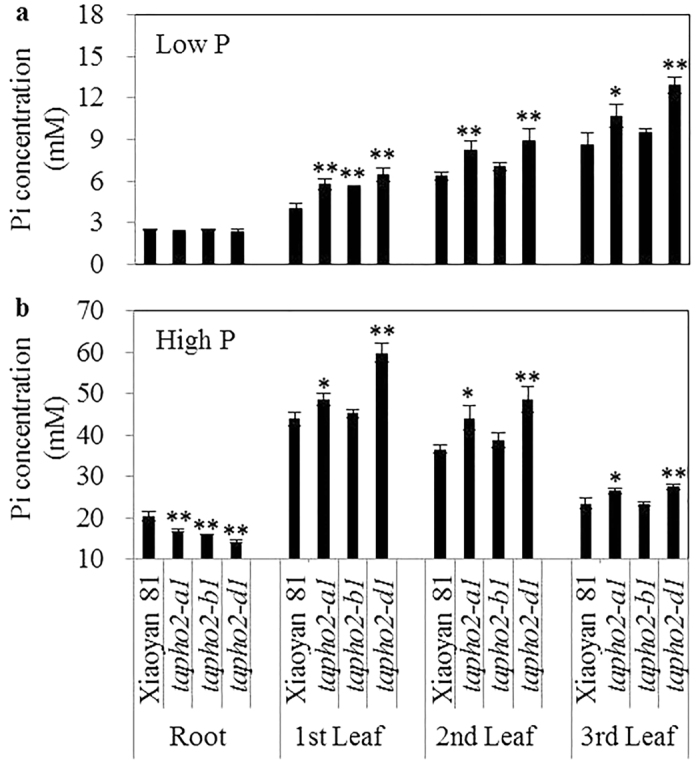
Pi concentrations in roots and expanded leaves of the wild type and *tapho2* mutant plants grown in nutrient solutions containing 10 μM Pi (low P) and 200 μM Pi (high P) levels at the seedling stage. (**a**,**b**) Pi concentrations in roots and different age leaves of the plants grown in low P (**a**) and high P (**b**) nutrient solutions. Error bars indicate SE (n = 5). Asterisks indicate the significance of differences between wild type and *tapho2* mutants as determined by Student’s *t* test analysis: **P < 0.01, *P < 0.05.

We next measured the Pi accumulation in roots and expanded leaves of the *tapho2* mutants and wild type. Low P treatment greatly reduced root and leaf Pi concentrations and altered Pi distributions in leaves, as compared to high P treatment (Fig. 5). The leaf Pi concentrations in the mutants and wild type decreased with leaf ages under low P conditions (Fig. 5a); in contrast, they increased with leaf ages under high P conditions (Fig. 5b). The *tapho2-a1* and *-d1* mutants had significantly higher Pi concentrations in all the examined leaves under both low P and high P conditions, and had significantly lower Pi concentrations in roots under high P conditions than did the wild type (Fig. 5). The *tapho2-b1* mutant had significantly higher Pi concentrations in the 1^st^ leaf under low P conditions (Fig. 5a), and significantly lower Pi concentrations in roots under high P conditions than did the wild type (Fig. 5b). After comparing the leaf Pi concentrations in the mutants and wild type, we found that *tapho2-d1* had the strongest, while *tapho2-b1* had the weakest effects on leaf Pi concentrations under both low P and high P conditions (Fig. 5).

**Figure 6:**
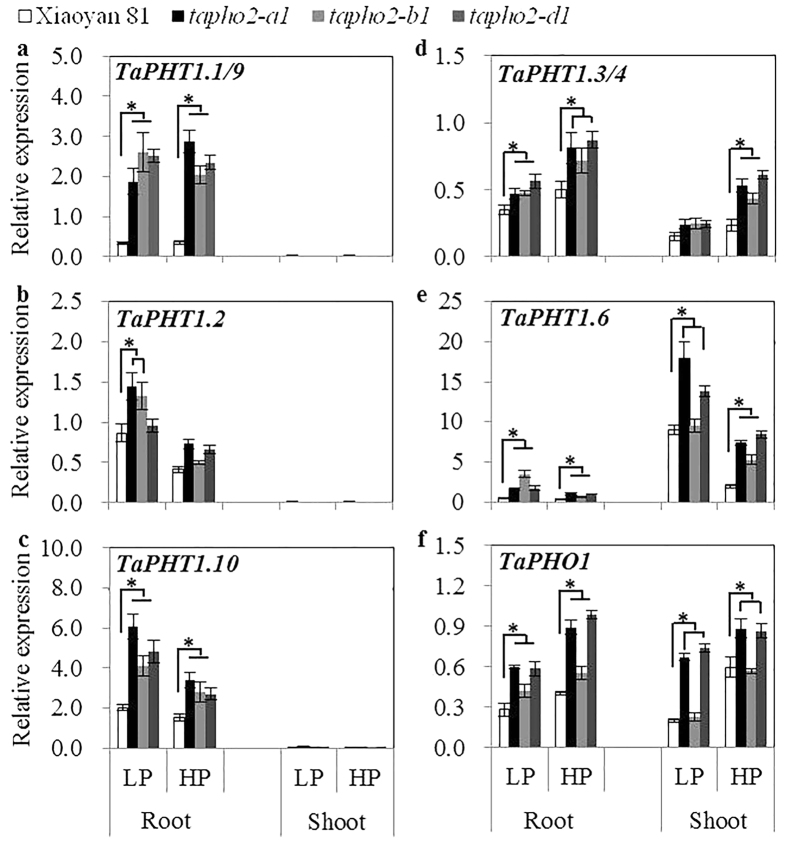
Expression levels of *TaPHT1s* and *TaPHO1* in roots and shoots of the wild type and *tapho2* mutant plants grown in nutrient solutions containing 10 μM Pi (low P) and 200 μM Pi (high P) levels. (**a**) *TaPHT1.1/1.9*; (**b**) *TaPHT1.2*; (**c**) *TaPHT1.10*; (**d**) *TaPHT1.3/4*; (**e**) *TaPHT1.6*; (**f**) *TaPHO1*. LP and HP indicate low P and high P treatment, respectively. Error bars indicate SE (n = 5). Asterisks indicate the significance of differences between wild type and *tapho2* mutants as determined by Student’s *t* test analysis: *P < 0.05.

To understand the possible mechanisms that *tapho2* mutants affected Pi distribution, we analyzed the expression of *PHT1* and *PHO1* transporters in roots and shoots. All the *tapho2-a1, -b1* and *-d1* mutants had higher expression of *TaPHT1* transporters under low P or high P conditions than did the wild type (Fig. 6a–e). The *tapho2-a1* and *-d1* mutants had higher expression of *TaPHO1* than did the wild type, but the *tapho2-b1* mutants showed similar expression of *TaPHO1* with the wild type under both low P and high P conditions (Fig. 6f).

### Effects of deleting *TaPHO2* on wheat growth and P uptake in field experiments

**Figure 7:**
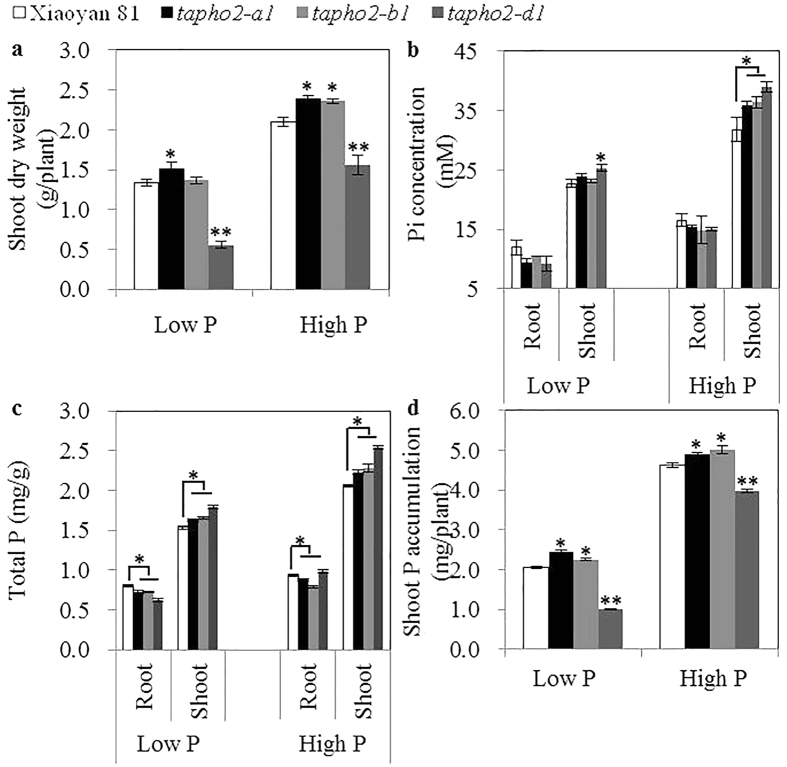
Shoot dry weight and P content of the wild type and *tapho2* mutant plants grown under 0 g P m^−2^ (low P) and 16 g P m^−2^ (high P) conditions at seedling stage in the field experiment of 2014–2015 growing season. (**a**) Shoot dry weight. (**b**) Pi concentration. (**c**) Total P concentration. (**d**) Total P accumulated in shoots. Error bars indicate SE (n = 5). Asterisks indicate the significance of differences between wild type and *tapho2* mutants as determined by Student’s *t* test analysis: **P < 0.01,*P < 0.05.

We measured SDW of the *tapho2* mutants and wild type at seedling stage under low P and high P conditions in the field experiment of 2014–2015 growing season. Compared to the wild type plants, the *tapho2-a1* mutant had significantly higher SDW under both low P and high P conditions, the *tapho2-b1* mutant had significantly higher SDW under high P conditions, and the *tapho2-d1* mutant had significantly lower SDW under both low P and high P conditions (Fig. 7a). We next analyzed Pi and total P concentrations in roots and shoots. These three mutants all had significant lower Pi and total P concentrations in roots than did the wild type under low P conditions, and had significant higher Pi and total P concentrations in shoots than did the wild type under both low P and high P conditions (Fig. 7b,c). These results indicated that the translocation of P from roots to shoots was increased in the *tapho2* mutants. Calculation of aerial P accumulations (total P accumulated in shoots) revealed that the *tapho2-a1* and *-b1* mutants had significantly higher aerial P accumulations than did the wild type both under low P and high P conditions; In contrast, *tapho2-d1* mutant had significantly lower aerial P accumulations than did the wild type under both low P and high P conditions (Fig. 7d).

We then investigated the agronomic traits at maturity in two consecutive field experiments. In both the 2013–2014 and 2014–2015 growing seasons, the *tapho2-a1* mutant had significantly higher biomass yield and grain yield than did the wild type under low P conditions, and the *tapho2-d1* mutant had significantly lower biomass yield and grain yield than did the wild type under both low P and high P conditions (Table 1). In the 2014–2015 growing season, we observed that *tapho2-d1* mutant had significantly shorter plant height than did the wild type under both low P and high conditions (Table 1).

The P-use related traits were also analyzed in the 2014–2015 growing season. All the three *tapho2* mutants had higher total P concentration in grains than did the wild type under both low P and high P conditions (Table 1). Under both low P and high P conditions, the total P accumulated in the aerial parts in the *tapho2-a1* and *tapho2-b1* mutants were significantly higher than that in the wild type; in contrast, those in the *tapho2-d1* mutant were significantly lower than that in the wild type (Table 1).

## Discussion

The crucial roles of *PHO2* in regulating Pi signaling have been elaborately depicted in Arabidopsis and rice, and *PHO2* exists in single copy in these diploid plant species^12^,^13^,^16^. As common wheat is an allohexaploid which contains three homoeologous genomes^28^, theoretically it may have three *PHO2* homologous genes. In the wheat variety Xiaoyan 81, we isolated *TaPHO2-A1, TaPHO2-B1* and *TaPHO2-D1* on chromosomes 1A, 1B and 1D, respectively (Fig. 1A and Supplemental Fig. S1). These three wheat genes seemed to be the orthologues of the *PHO2* genes in Arabidopsis and rice. Firstly, the deduced TaPHO2 protein sequences closely related to AtPHO2 and OsPHO2 in the phylogenetic tree of PHO2 proteins (Supplemental Fig. S3), and had conserved UBCc domain (Supplemental Fig. S2). Secondly, all the three *TaPHO2s* conferred five putative miR399-binding sites in the 5′-UTR (Supplemental Fig. S4), and were able to be degraded by tae-miR399 (Fig. 2), as has been shown in Arabidopsis and rice^12^,^13^,^16^. The degradation of the *TaPHO2s* by tae-miR399-A1 was able to be inhibited by *TaIPS1.1* (Fig. 2). Previously, the miR399 activity in cleaving *PHO2* has been found to be reduced by *IPS1* according to the target mimicry mechanism in Arabidopsis^14^. As such, the *IPS1*-miR399-*PHO2* signaling cascade is conserved in plants. Finally, knockout mutants of the three *TaPHO2* genes displayed increased Pi translocation from roots to shoots and leaf Pi concentrations (Fig. 5). PHO2 has been demonstrated to regulate PHO1 and PHT1 transporters at post-translational level^17^,^18^. As such, further research is needed to explore the PHO1 and PHT1 transporters which may contribute to the increased Pi translocation from roots to shoots and leaf Pi concentrations in the *tapho2* mutants. In present study, although we did not investigate the protein abundance of PHO1 and PHT1 transporters in the mutants and wild type plants, we found that the *tapho2* mutants had higher expression of *TaPHT1* and *TaPHO1* transporters than the wild type (Fig. 6). Similar results have been reported in Arabidopsis and rice that loss of function of *PHO2* has increased expression of *PHO1* and *PHT1* transporters^12^,^13^,^16^,^17^,^18^.

The three *TaPHO2* genes shared similar gene structure, highly similarity in ORF sequences and deduced protein sequences (Fig. 1a and Supplemental Fig. S2), and similar response to Pi-deficiency (Fig. 1c), but they exhibited large difference in expression levels (Fig. 1c). In both roots and shoots, *TaPHO2-D1* exhibited much higher transcript abundance than did *TaPHO2-A1* and *-B1*, and *TaPHO2-B1* had the lowest expression among these three *TaPHO2* genes (Fig. 1c). In consist with these results, the overall expression of *TaPHO2* in roots of the *tapho2-a1, -b1* and *-d1* mutants was reduced to 55.8%, 70.1% and 21.1% of the wild type level (Fig. 3a), respectively. Among the *tapho2-a1, -b1* and *-d1* mutants, *tapho2-d1* had the strongest, *tapho2-a1* the moderate, and *tapho2-b1* the weakest phenotype in term of leaf Pi concentration under both low P and high P conditions when the wheat seedlings were grown hydroponically (Fig. 5). In the field experiment of 2014–2015 growing season, the *tapho2-d1* mutant had substantially higher Pi and total P concentrations in shoots at seedling stage (Fig. 7b,c), and total P concentration in grains at maturity than did the *tapho2-a1* and *-b1* mutants, and the wild type as well (Table 1). Taking information together, Pi and total P concentrations in leaves and grains negatively correlated with the expression of *TaPHO2*. These negative correlations suggested that the increased Pi and total P concentrations in leaves and grains mainly resulted from the deletion of *TaPHO2*, although some other genes surrounding the interested gene might be deleted in the mutants when the mutation was induced by ion beam^29^.

In diploid plant species such as Arabidopsis and rice, loss of function of *PHO2* has been found to inhibit plant growth, possibly caused by the over-accumulation of Pi in shoots^13^,^16^. These results suggest that *PHO2* is essential to maintain Pi homeostasis and hence plant growth. Our present study found that a severe reduction in *PHO2* expression could also impair Pi homeostasis and plant growth in wheat. Although wheat contains three expressed *TaPHO2* genes, the overall expression of *TaPHO2* was severely reduced (only 21.1% of the wild type level) when *TaPHO2-D1* was deleted in the *tapho2-d1* mutant (Fig. 3a). Compared to the wild type plants, the *tapho2-d1* mutant displayed inhibited growth at seedling stage (Figs 4c,d and 7a) and at maturity (Table 1), and increased Pi and total P concentrations in leaves and grains (Figs 5 and 7 and Table 1). As such, the *tapho2-d1* mutant resembled the phenotypes of *pho2* mutants in Arabidopsis and rice. In contrast, the phenotypes of the *tapho2-a1* mutant suggested that a moderate reduction in *TaPHO2* expression might improve P uptake and grain yield in wheat. Compared to the wild type and *tapho2-d1* mutant, Pi and total P concentrations in aerial parts (leaves, shoots and grains) were moderately increased (Figs 5 and 7 and Table 1), these moderately increased P accumulation might benefit to achieve higher grain yield under low P conditions (Table 1). Although both *tapho2-a1* and *tapho2-d1* mutant had increased leaf Pi concentration (Fig. 5), they showed opposite effects on yield and biomass (Table 1). Furthermore, the shoot growth of *tapho2-d1* mutant was inhibited under low P conditions (Fig. 4c); even though the leaf concentrations in the *tapho2-d1* mutant under low P conditions were lower than those of the wild type plants under high P conditions (Fig. 5). These results may suggest a role of *TaPHO2-D1* in regulating plant development besides its function in controlling Pi homeostasis. Indeed, it has been reported that the miR399-*PHO2* module regulates the flowering time in response to different ambient temperatures in Arabidopsis^30^.

In summary, we identified three *TaPHO2* genes from the homoeologous group 1 of the hexaploid wheat. They displayed large difference in transcription abundance, and their knock out mutations exerted different effects on P uptake and distribution, and plant growth. Phenotype evaluation in the three *tapho2* mutants and wild type revealed the negative correlation between Pi and total P concentrations in aerial parts and *TaPHO2* expression levels. A moderate reduction in *PHO2* expression by deleting *TaPHO2-A1* improved P uptake and grain yield under low P conditions. Our founding provided useful cue to increase wheat yield with less P fertilizer input through conventional breeding by using *tapho2-a1* mutant as a parent, or engineering *PHO2* expression level by genome editing and RNA interference (RNAi) approach.

## Materials and Methods

### Plant materials and growth conditions

The wild type and *tapho2* mutants were used in this work. The mutants were screened from Xiaoyan 81 deletion mutant library irradiated by nitrogen ions^29^. Xiaoyan 81 is a winter wheat (*Triticum aestivum*) variety commercially released in 2006.

Hydroponic culture and field experiments were used to evaluated the phenotypes of the wild type and mutants. For hydroponic culture, the nutrient solution composition, methods for seed sterilization and germination, and the growth conditions for the hydroponic experiments were described previously^31^. Briefly, the plants were grown in a growth chamber with 20 ± 1 °C, 50% to 70% relative humidity, a photon fluence rate of 300 mmol photons m^−2^ s^−1^, and a 16 h day/8 h night cycle conditions. The nutrient solution contained 200 μM KH_2_PO_4_ was used as high P treatment, and the low P treatment was substituted with 10 μM KH_2_PO_4_ and 190 μM KCl. The nutrient solution was adjusted to pH 6.0 by 1 M HCl, and refreshed every 2 days.

Two consecutive field experiments were conducted in the Beijing experiment station of the IGDB in 2013–2014 and 2014–2015 growing seasons. The experiments consisted of two treatments and a random block design with five replications was used. The high P treatment was applied 16 g P m^−2^ in the form of calcium superphosphate prior to sowing and the low P treatment had no P application. Both treatments had 18.0 g m^−2^ of N in the form of urea with 12 g m^−2^ applied prior to sowing and 6 g m^−2^ applied at the stem elongation stage. The seeds were sown in the end of September at a seed rate of 108.7 seeds m^−2^, and the plants were harvested in the middle of the following June. Each genotype in each replication had two 1.5 m long rows spaced with 23 cm.

### Gene cloning and phylogenetic analysis

The sequence of *AtPHO2* (AT2G33770) was used as query to search the wheat ESTs data base of NCBI. Two pairs of conserved primers (Supplementary Table S1) were designed for the 5′ and 3′ RACE PCR amplification according to the identified ESTs. First-strand cDNA synthesis and RACE PCR were performed with the SMARTer^TM^ RACE cDNA Amplification Kit (Clontech) following the instructions. Thereafter, three full-length of sequences, named as *TaPHO2-A1, TaPHO2-B1* and *TaPHO2-D1* respectively, were further isolated from cultivar Xiaoyan 81. The gene structures of *TaPHO2s* were aligned by using ClustalX 2.0^32^. The protein structures and conserved ubiquitin-conjugating catalytic domain were analyzed by CCD analysis (http://www.ncbi.nlm.nih.gov/Structure/cdd/wrpsb.cgi). The phylogenetic analysis was performed by neighbor-joining method, and the phylogenetic tree was drawn with MEGA 5.0^33^.

### *TaPHO2* deletions screening

Conserved forward and reverse primers were designed according to the differences in the seventh intron of the three homologous *TaPHO2* genes. This primer set (Supplementary Table S1) amplified three fragments product (with different sizes) in genomic PCR, which were specific for the *TaPHO2-A1* (424 bp), *TaPHO2-B1* (412 bp) and *TaPHO2-D1* (397 bp) of Xiaoyan 81, respectively. For facilitative screening, the forward primer was labeled with 6-FAM fluorophore at the 5′ terminus. PCR was performed in a volume of 20 μL containing 100 ng genomic DNA, 10 μM of each primer, 2 × Taq Mix (GenStar). The amplification program starting at 94 °C 3 min, followed by 35 cycles of 94 °C 30 s, 60 °C 25 s, 72 °C 40 s, with a final extension at 72 °C 5 min. The resulted PCR products were separated by ABI 3730 analysis, and the *TaPHO2* deletion mutant was confirmed based on the absence of the corresponding fragment. The *tapho2* mutants were backcrossed two times with Xiaoyan 81 as recurrent parent. Homozygous *tapho2-a1, b1* and *d1* deletion lines were obtained from BC_2_F_2_.

### Quantitative RT-PCR analysis

Total RNA was extracted with TRIzol reagent (Ambion). Reverse transcription was performed with the ReverTra Ace qPCR RT Master Mix with gDNA Remover (Toyobo) using 1 μg total RNA. Quantitative RT-PCR analysis was carried out with a LightCycler 480 engine (Roche) by using the LightCycler480 SYBR Green I Master Mix (Roche). The relative transcript level of each cDNA sample was calculated from triplicates using the formulate 2^−ΔCt^ after normalization to *TaACTIN* (in common wheat) or *NtACTIN* (in tobacco) control. The primers used for qRT-PCR analysis were listed in Supplementary Table S2. As the three *TaPHO2* homologous genes displayed highly sequence similarities, it is difficult to design gene specific primer pair in the similar regions. The designed three pairs of gene specific primers for *TaPHO2-A1, TaPHO2-B1* and *TaPHO2-D1* located at 1281~1462 bp, −215~−58 bp (spanning the last two miR399 binding sites) and 2377~2521 bp (the ATG start codon defined as 1), respectively. The primer pair for detecting the overall expression of *TaPHO2* located at 2220~2341 bp (relative to the ATG start codon of *TaPHO-A1*).

### Vector construction and plant transformation

To investigate the regulation of tae-miR399 and *TaIPS1* on *TaPHO2* degradation, a 1.6 Kb cDNA fragment containing the five putative miR399 binding sites of the three *TaPHO2* genes were cloned into pRI101-AN vector (Takara). The full length of tae-miR399 and *TaIPS1.1* were also cloned into pRI101-AN vector. All the resulting binary vectors were introduced into the GV3101 strain of *Agrobacterium tumefaciens*. The *Agrobacterium* mediated transient expression assays in tobacco leaves were conducted as described by Liu *et al*.^17^. The Primers used for vector construction were listed in Supplementary Table S1.

### Measurements of P concentration

Plant samples were frozen after measured fresh weight or dried at 80 °C for 3 days to a constant dry weight. The Pi and total P concentrations were measured using the method described by Wang *et al*.^34^ with some modified. For Pi concentration measurement, a frozen sample was homogenized with 400 μL of 5 M H_2_SO_4_ and 4 mL H_2_O, using an ice-cold mortar and pestle. After the mixture was centrifuged at 12000 g for 10 min at 4 °C, the supernatant was diluted appropriately and carried out with a SmartChem200 analyzer (Alliance) by following the instruction of P analysis. Pi concentration was calculated by normalization of fresh weight.

For total P concentration measurement, the dried plant samples (about 0.2 g) were milled and subsequently digested with 5 mL H_2_SO_4_. The digested solution was cooled and diluted to 100 mL. The solution was diluted appropriately and Pi content was analyzed as described above. The total P content was calculated by normalization of dry weight.

### Statistical analysis

One-way ANOVA was performed with SPSS19 for mean comparisons between the wild type and mutant plants.

## Additional Information

**How to cite this article**: Ouyang, X. *et al*. Knock out of the *PHOSPHATE 2* Gene *TaPHO2-A1* Improves Phosphorus Uptake and Grain Yield under Low Phosphorus Conditions in Common Wheat. *Sci. Rep.* **6**, 29850; doi: 10.1038/srep29850 (2016).

## Supplementary Material

Supplementary Information

## Figures and Tables

**Table 1:** Agronomic traits and P uptake of the wild type and *tapho2* mutant plants grown under low (0 g P m^−2^) and high (16 g P m^−2^) P conditions in the field experiments.

**Trait**	**Low P**				**High P**			
	**Xiaoyan 81**	***tapho2-a1***	***tapho2-b1***	***tapho2-d1***	**Xiaoyan 81**	***tapho2-a1***	***tapho2-b1***	***tapho2-d1***
2013–2014								
Biomass (g/plant)	20.27 ± 1.11	22.91 ± 0.76*	21.06 ± 1.55	13.85 ± 1.56**	27.60 ± 0.29	29.18 ± 0.86	27.88 ± 1.23	18.41 ± 0.84**
GY (g/plant)	8.02 ± 0.66	9.17 ± 0.30*	8.38 ± 0.23	4.77 ± 0.31**	10.52 ± 0.43	12.53 ± 0.65**	11.33 ± 0.44	6.85 ± 0.13**
SN	6.31 ± 0.54	6.40 ± 0.18	6.25 ± 0.14	5.51 ± 0.53	8.85 ± 0.13	9.63 ± 0.23	8.70 ± 0.79	7.65 ± 0.12*
GN	32.85 ± 1.49	34.75 ± 1.29	34.33 ± 0.99	24.00 ± 0.12**	31.06 ± 0.65	32.80 ± 3.04	31.87 ± 0.78	25.55 ± 0.60**
TGW (g)	38.88 ± 0.91	41.29 ± 0.59*	39.05 ± 0.56	36.33 ± 1.16	37.78 ± 0.82	38.08 ± 0.99	37.60 ± 0.86	35.23 ± 0.65*
2014–2015								
PHT (cm)	61.2 ± 0.8	58.8 ± 0.6	61.4 ± 0.8	53.2 ± 1.2**	67.8 ± 0.4	68.2 ± 1.2	69.8 ± 0.6	61.8 ± 1.3**
Biomass (g/plant)	13.07 ± 0.27	14.50 ± 0.54*	14.35 ± 0.47*	9.41 ± 0.30**	23.21 ± 1.32	24.16 ± 1.03	23.50 ± 1.02	17.39 ± 1.13**
GY (g/plant)	5.35 ± 0.18	6.27 ± 0.30*	5.90 ± 0.28	3.27 ± 0.18**	10.30 ± 0.65	11.22 ± 0.43	10.92 ± 0.38	7.51 ± 0.47**
SN	5.48 ± 0.24	5.50 ± 0.13	5.52 ± 0.28	4.72 ± 0.15*	9.44 ± 0.55	9.42 ± 0.54	9.24 ± 0.31	8.64 ± 0.29
GN	41.25 ± 0.95	43.03 ± 1.37*	41.88 ± 0.91	32.47 ± 0.96**	39.52 ± 1.86	43.56 ± 0.87	42.03 ± 0.65	31.53 ± 0.46**
TGW (g)	37.84 ± 0.48	39.86 ± 0.53*	36.95 ± 0.28	33.49 ± 0.59**	37.29 ± 0.41	37.68 ± 0.37	37.56 ± 0.32	35.38 ± 0.36**
GPC (mg/g)	2.44 ± 0.05	2.66 ± 0.06**	2.69 ± 0.03**	3.19 ± 0.04**	2.72 ± 0.05	2.88 ± 0.06*	3.00 ± 0.05**	3.48 ± 0.03**
SPC (mg/g)	0.20 ± 0.01	0.32 ± 0.07	0.20 ± 0.02	0.28 ± 0.01	0.22 ± 0.01	0.33 ± 0.05	0.28 ± 0.01	0.33 ± 0.04
APA (mg/plant)	14.75 ± 0.49	18.38 ± 0.64**	17.71 ± 0.33**	12.39 ± 0.22**	31.62 ± 0.91	35.72 ± 1.11**	38.05 ± 0.96**	29.85 ± 0.44*
PHI	0.895 ± 0.003	0.857 ± 0.005**	0.904 ± 0.002**	0.860 ± 0.003**	0.909 ± 0.003	0.881 ± 0.003**	0.883 ± 0.003**	0.890 ± 0.002**

PHT, Plant height. GY, Grain yield per plant. SN, Spike number per plant. GN, Grain number per spike. TGW, thousand-grain weight. GPC, Grain total P concentration. SPC, Straw total P concentration. APA, total P accumulated in aerial parts. PHI, phosphorus harvest index. Data are the mean ± SE (n = 5). Asterisks indicate the significance of differences between wild type and *tapho2* mutant plants as determined by Student’s t test analysis: **P < 0.01,*0.01 < P< 0.05.
