# Supplementary material for: Knock out of the PHOSPHATE 2 Gene TaPHO2-A1 Improves Phosphorus Uptake and Grain Yield under Low Phosphorus Conditions in Common Wheat
Source: Sci Rep. 2016 Jul 15;6:29850. doi: 10.1038/srep29850 (PMC4945926; doi:10.1038/srep29850)
Supplement: Supplementary Information [file srep29850-s1.pdf]

Knock out of the *PHOSPHATE 2* Gene *TaPHO2-A1* Improves Phosphorus Uptake and Grain Yield under Low Phosphorus Conditions in Common Wheat

Xiang Ouyang [1], Xia Hong [1,2], Xueqiang Zhao[1], Wei Zhang [1], Xue He [1], Wenying Ma [1], Wan Teng [1], Yiping Tong [1,*]

[1] State Key Laboratory for Plant Cell and Chromosome Engineering, Institute of Genetics and Developmental Sciences, Chinese Academy of Sciences, Beijing 100101, China

[2] Taizhou Academy of Agricultural Sciences, Linhai, Zhejiang 317000, China

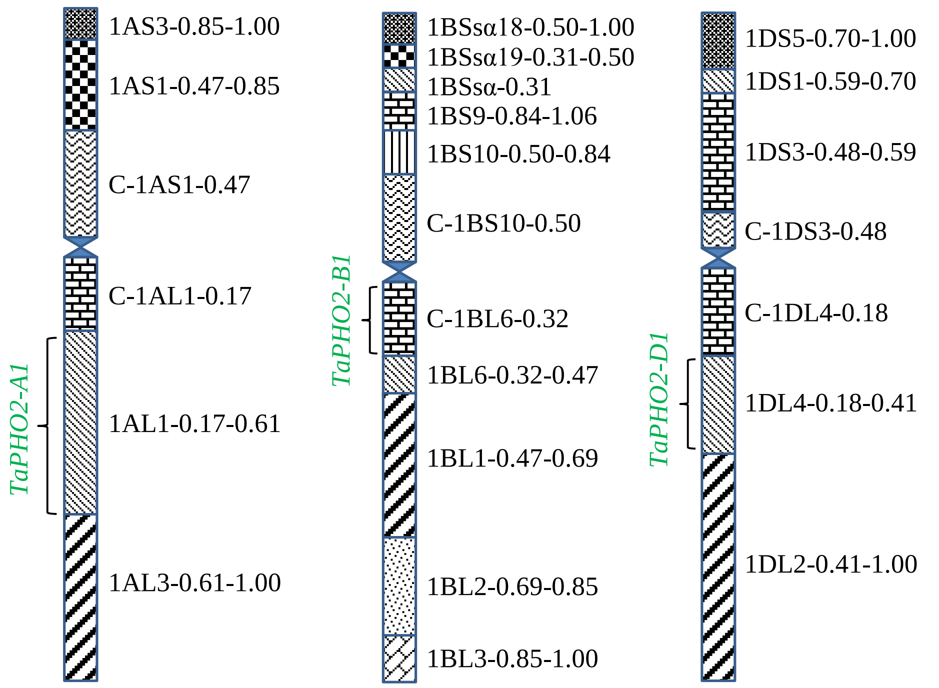

Figure S1. **Chromosome localizations of *TaPHO2*.** The *TaPHO2-A1*, *TaPHO2-B1* and *TaPHO2-D1* were located at 1AL1-0.17-0.61 on chromosome 1A, C-1BL6-0.32 on chromosome 1B, and 1DL4-0.18 on chromosome 1D, respectively.

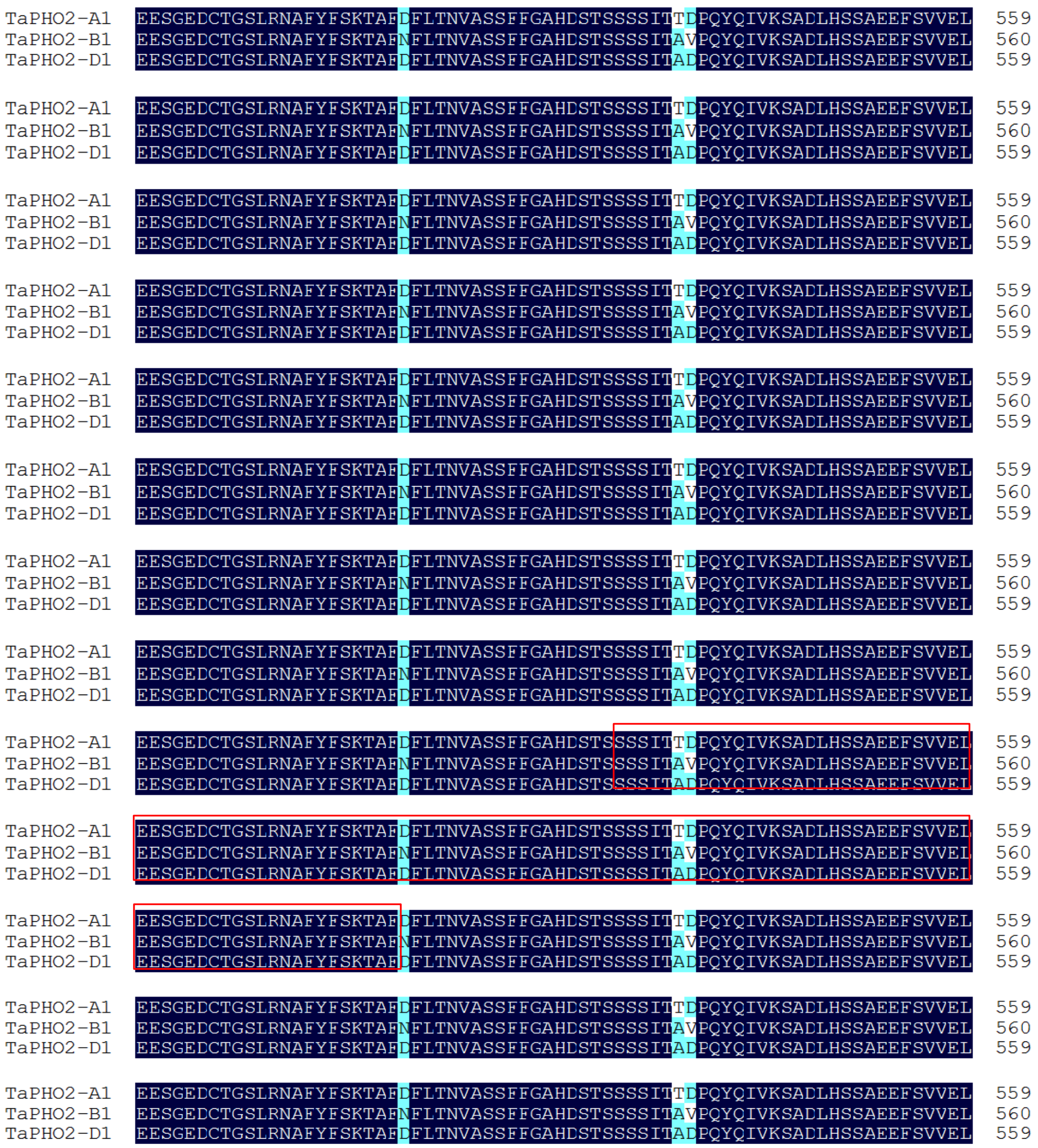

Figure S2. **The deduced amino acid sequences of *TaPHO2-A1*, *B1* and *D1*.** The red boxes indicate the position of the conserved ubiquitin-conjugating enzyme E2 catalytic (UBCc) domain.

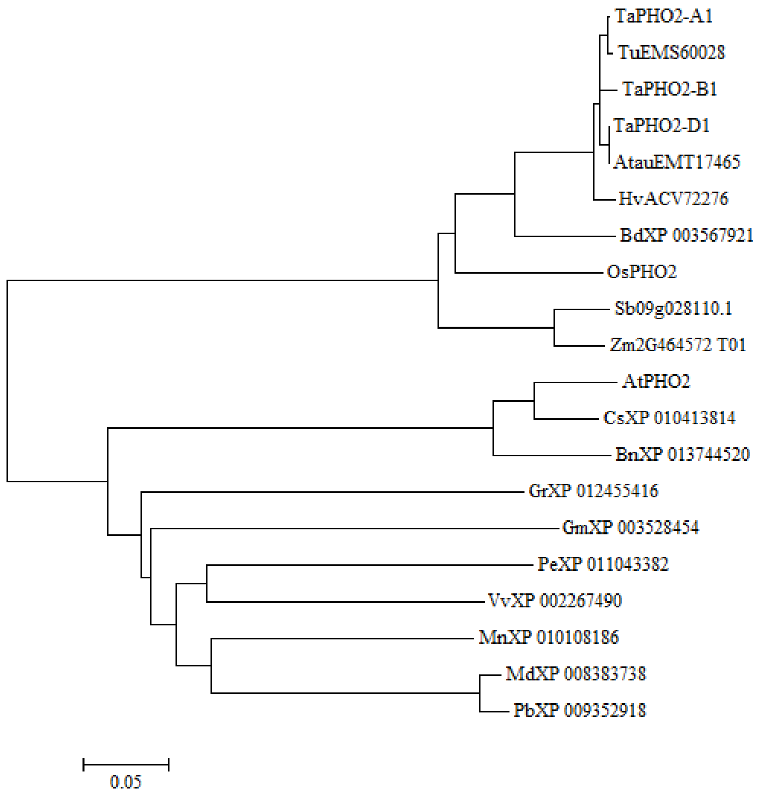

Figure S3. **Phylogenetic analysis of PHO2 proteins.** Unrooted phylogenetic tree of the PHO2 from *Triticum urartu* (Tu), *Aegilops tauschii* (Atau), *Hordeum vulgare* (Hv), *Brachypodium sylvaticum* (Bd), *Oryza sativa* (Os), *Sorghum bicolor* (Sb), *Zea Mays* (Zm), *Arabidopsis thaliana* (At), *Camelina sativa* (Cs), *Brassica napus* (Bn), *Gossypium raimondii* (Gr), *Glycine max* (Gm), *Populus euphratica* (Pe), *Vitis vinifera* (Vv), *Morus notabilis* (Mn), *Malus domestica* (Md), *Pyrus x bretschneideri* (Pb). The tree was made using the neighbor-joining method. Protein accession numbers are: OsPHO2, BAH01100; AtPHO2, NP_850218.

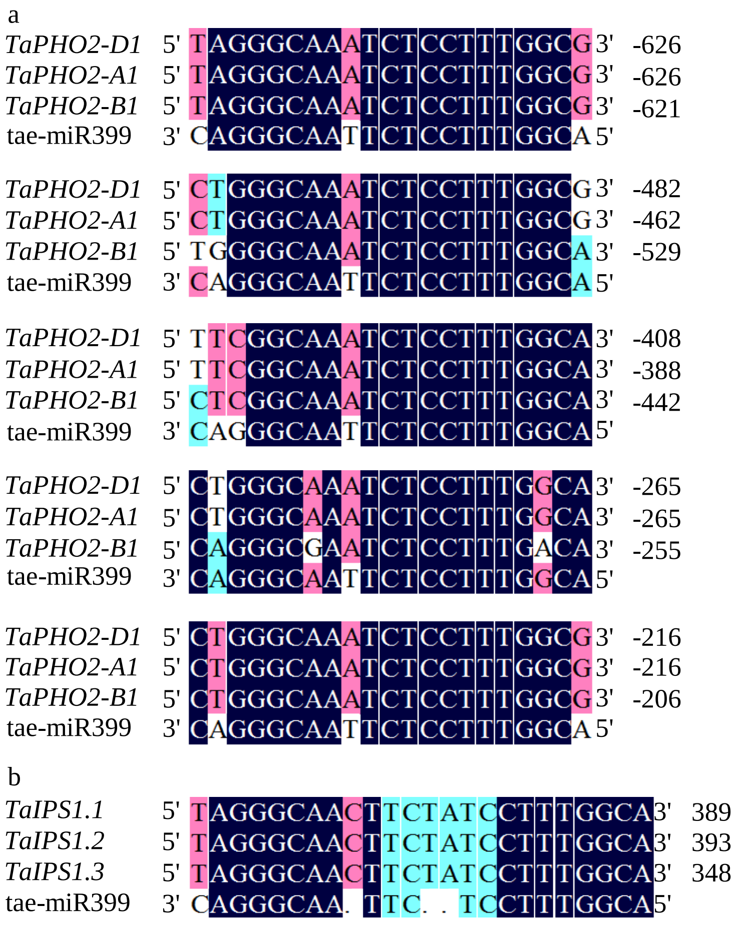

Figure S4. The putative miR399 binding sites in the 5′-UTR region of *TaPHO2* (a) and *TaIPS1* (b). The target sites of *TaPHO2* and *TaIPS1* genes were aligned with tae-miR399 (MI0006176) mature sequence with DNAMAN6.0.

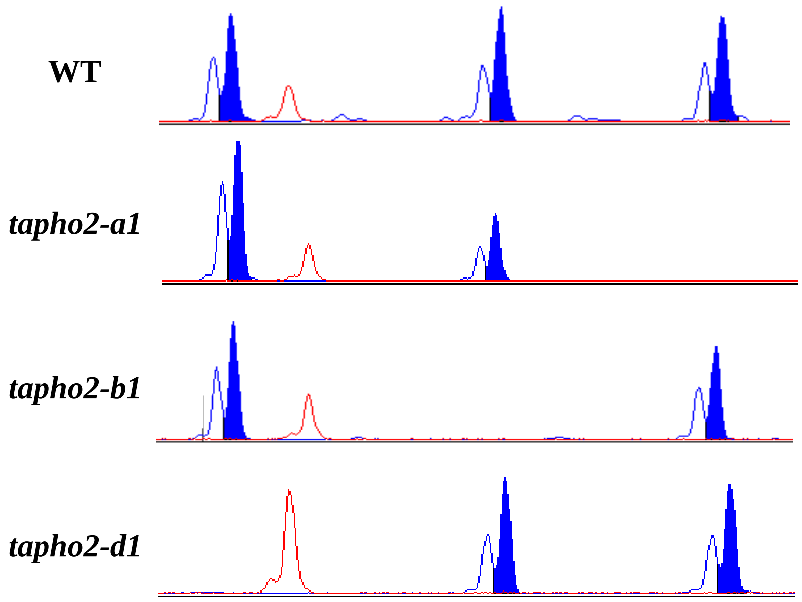

Figure S5. **The screening results of *tapho2* deletion mutants by ABI 3730 analysis**. The specific forward and reverse primers were used by simultaneously amplifying the three homologous *TaPHO2* genes with length polymorphisms.

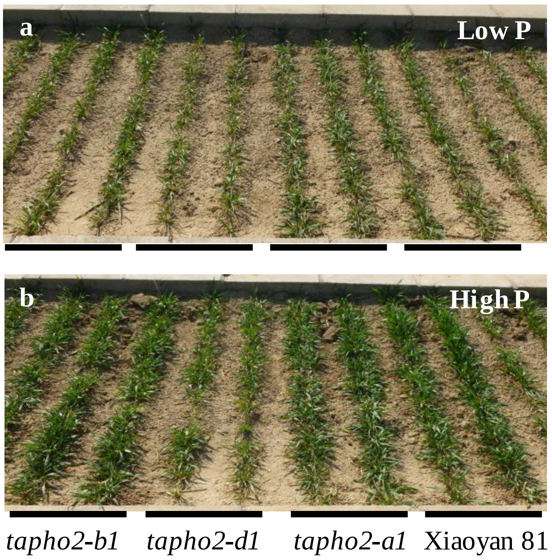

Figure S6. **Growth performance of *tapho2* mutants grown under 0 g P m^{-2} (low P) and 16 g P m^{-2} (high P) conditions at seedling stage in the field experiment 2014-2015 growing season**. (a) Low P treatment. (b) High P treatment.

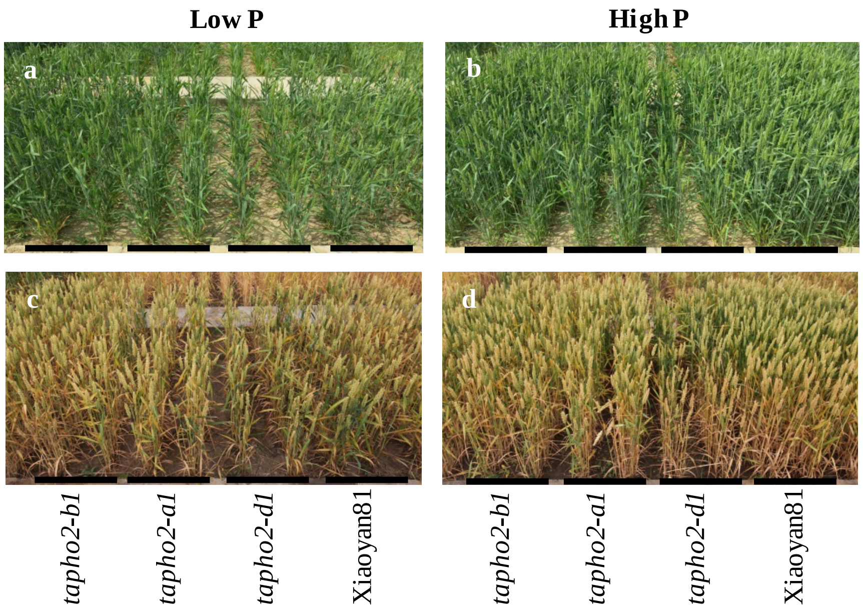

Figure S7. **Growth performance of *tapho2* mutants grown under 0 g P m^{-2} (low P) and 16 g P m^{-2} (high P) conditions in the field experiment of 2014-2015 growing season**. (a, b) Flowering stage. (c, d) 28 days after flowering.

Table S1. Primers used for RACE PCR, deletion mutants screening and plasmid constructs in this work.

Gene	Primer Name	Primer Sequence (5'-3')
TaPHO2	TaPHO2-5'-RACE-R1	TGCTTCCCTTATGCTAACTGGCAGTTGG
	TaPHO2-5'-RACE-R2	CACATCAACATGAGACACAACACCTTC
	TaPHO2-3'-RACE-F1	CTTGCTGTCTTGCAAGTCCATGATG
	TaPHO2-3'-RACE-F2	AGCACGTACATATCAGATGATGGCT
TaPHO2	TaPHO2-Mutant-F1	GTGAACAGAACATGCGTACTCTTGTG
	TaPHO2-Mutant-R2	AGGTCCATCCTTTCCTCATAAACTCT
TaPHO2-A1	TaPHO2-A-HuZ-F1	CGTCATATGTCTCTCTACGTCTCTCGG
TaPHO2-B1	TaPHO2-B-HuZ-F1	CACATATGCCAGCCCTCCGTGTCC
TaPHO2-D1	TaPHO2-D-HuZ-F1	CCATATGCGCGCTCTCTCTTTCTCG
	TaPHO2-HuZ-R2	GAGGAATTCACAATCCTCACGCGTTGA
tae-miR399	taemiR399-HuZ-F1	GAAGCATATGCCATCCGGAGATCCA
	taemiR399-HuZ-R2	GTCGACGCACCTATGAGGCTGCATGGTCTTC
TaIPS1.1	TaIPS1.1-HuZ-F1	ACATATGCCACAAGATCTCAGATAGC
	TaIPS1.1-HuZ-R2	AGAATTCACACACACCCATGCTAGTTA

Table S2. Primers used for Real time RT-PCR analysis in this work.

Gene	Primer Name	Primer Sequence (5'-3')
TaPHO2	TaPHO2-Real-F1	GGAGAAGAACTCCATCACGTACAACG
	TaPHO2-Real-R2	GGCAAGTGAAGTGCTCCTTGACGA
TaPHO2-A1	TaPHO2-Real-1A-F1	GTATAAGGATGATGGAATTGAAGTA
	TaPHO2-Real-1A-R2	CATTCTTAGTACTCTCATGGTGAT
TaPHO2-B1	TaPHO2-Real-1B-F1	GGTTTAGCTTCAGTCCTGTCAG
	TaPHO2-Real-1B-R2	CAGCCTTTGAACAGCGGTC
TaPHO2-D1	TaPHO2-Real-1D-F1	CTCGGCGGTGATCTCATTG
	TaPHO2-Real-1D-R2	AGGCGATCCCAGCTTCGC
TaPHO1	TaPHO1-Real-F1	GAGTGGCTACCACAAATTGAATC
	TaPHO1-Real-R2	TATTTTTACATCCATGTCAAAGGTG
TaPHT1.1	TaPHT1.1-Real-F1	GAGACCGGCTACTCACGGG
	TaPHT1.1-Real-R2	CTAAGCTTCGATGCCATCGTC
TaPHT1.2	TaPHT1.2-Real-F1	TGAGACCGGCTACTCACGG
	TaPHT1.2-Real-R2	AATGGAGTCATCGTCGCCAA
TaPHT1.6	TaPHT1.6-Real-F1	TTTTTTATGGTCGGAGAGCGTT
	TaPHT1.6-Real-R2	CAGCCCTAATTAACCTGGACAACT
TaPHT1.7	TaPHT1.7-Real-F1	GTGTACGGCATGACGCTGAT
	TaPHT1.7-Real-R2	CCGAGCCAGAACCTGAAGAA
TaPHT1.10	TaPHT1.10-Real-F1	GCGTTCGGGTTCCTGTATGC
	TaPHT1.10-Real-R2	CAGTCGGAGCAATGGTGTCGT
TaActin	TaActin-Real-F1	ACCTTCAGTTGCCCAGCAAT
	TaActin-Real-R2	CAGAGTCGAGCACAATACCAGTTG
NtActin	NtActin-Real-F1	GATTTGCTGGTGATGATGCTCCTC
	NtActin-Real-R2	CCTTAGGATTAAGTGGTGCCTCAGT
TaPHO2	TaPHO2-NtReal-F1	CAGCAAGCAGCTTTCAAGAGTG
	TaPHO2-NtReal-R2	CACTTTGCCCCATCACTGAAC
TaIPS1	TaIPS1-NtReal-F1	CAGTACCAGCTGCATGCCTG
	TaIPS1-NtReal-R2	CTAGCCAACGCCGGATCCA
tae-miR399	taemiR399-NtReal-F1	GGAGGCATGCATGTACTGATG
	taemiR399-NtReal-R2	GGCAATTCTCCTTTGGCACG
